# Flexible ureteroscopy and laser lithotripsy for renal stones 2 cm or greater: A single institutional experience

**DOI:** 10.1097/MD.0000000000022704

**Published:** 2020-10-23

**Authors:** Jian-Sheng Huang, Jing Xie, Xiang-Jiang Huang, Qian Yuan, Hong-Tao Jiang, Ke-Feng Xiao

**Affiliations:** Department of Urology, Shenzhen People's Hospital, The Second Clinical Medical College of Jinan University, Shenzhen, China.

**Keywords:** flexible ureteroscopy, large kidney stone, laser, lithotripsy, stone-free rate

## Abstract

With improvements in endoscopy and laser technology, flexible ureteroscopy (FURS) has been a viable treatment option for large renal stones. Here, we share our experience of the FURS treatment for renal stones 2 cm or greater.

We evaluated 251 consecutive patients who underwent FURS and holmium laser lithotripsy for renal stones 2 cm or greater between January 2015 and April 2019. Stone size was defined as the longest axis on non-contrast computed tomography. Data were retrospectively collected from electronic medical records. Patient demographics, stone clearance rates and perioperative complications were evaluated.

There were 165 male patients and 86 female patients with an average age of 46.9 years (range 22–80 years). Mean stone size was 2.7 cm and the average number of procedures was 1.4 (range 1–5). The stone-free rate at the end of the first, second and third procedure was 61.9%, 82.9%, and 89.5%, respectively. The final stone-free rate decreased as stone size grows, and it was only 58.3% for kidney stones larger than 4 cm after an average of 2.3 procedures. The lowest clearance rates were observed in lower calyx calculi (87.2%) and multiple calyx calculi (83.5%). The overall complication rate was 15.1%, and the most common complication was postoperative fever (9.6%). One patient required blood transfusion, owing to postoperative coagulation disorders induced by urosepsis.

Single or staged FURS is a practical treatment option for the renal stones sized 2 to 4 cm with acceptable efficacy and safety. Stone clearance rate of FURS treatment is mainly affected by stone size and location.

## 1 Introduction

Currently, percutaneous nephrolithotomy (PCNL) remains the standard procedure for renal calculi greater than 2 cm in diameter.^[1]^ Despite the high clearance rate, there are non-negligible complications associated with PCNL, including fever in 10.8% of cases, transfusion in 7%, thoracic complication in 1.5%, sepsis in 0.5%, organ injury in 0.4%, embolization in 0.4%, and death in 0.05%.^[2]^ Although the “mini-PCNL” is applied with smaller access tracts in recent years, the complications rate remains high and up to 2% of cases require transfusions.^[3]^ In addition, PCNL is limited or contraindicated in patients with unfavorable characteristics, such as chronic anticoagulant therapy, morbidly obesity, and severe vertebral deformities.

With continuous improvement in endoscopy and laser technology, coupled with the increasing surgical experience, flexible ureteroscopy (FURS) has been a viable treatment option for large renal stones. Several studies have demonstrated successful FURS treatment of large renal calculi, with comparable stone-free rates (SFR) to PCNL and low complication rates.^[4,5]^ In this study, we present a single-center experience of the treatment of renal stones larger than 2 cm with FURS and holmium laser lithotripsy.

## 2 Methods

A total of 279 patients with renal stones greater than 2 cm in diameter were consecutively treated with FURS and holmium laser lithotripsy from January 2015 to April 2019, and data were retrospectively collected from electronic medical records. Patients aged below 18 years or with additional ureteral stones were excluded from this study. We did not exclude patients with multiple kidney stones, renal abnormalities, previous intervention with shock wave lithotripsy, endoscopic, or percutaneous treatment. Stone size was defined as the longest axis on non-contrast computed tomography, and patients with cumulative diameter greater than 2 cm of multiple stones were excluded. A total of 28 patients were excluded according to the above-mentioned criteria.

All patients received antibiotics therapy, most commonly as prophylaxis with cefuroxime (1500 mg bid), or adapted antibiotic in patients with positive urine culture. All procedures were performed by surgeons specialized in FURS treatment (who have at least 200 case experiences) using the 9.9F flexible ureteroscope (Olympus, Tokyo, Japan) through a 13/15F ureteral access sheath (Navigator, Boston Scientific, USA). Holmium laser lithotripsy was performed using 200 μm laser fibers, with laser energy and frequency of pulsation adjusted based on stone volume and density. Stone fragments were extracted using a 1.9-Fr nitinol basket (Escape, Boston Scientific, USA) and sent for analysis. A 5F double-J ureteral stent was routinely placed.

Residual stones were assessed with kidney ureter and bladder X-ray (KUB) or ultrasonography on postoperative day 1. If necessary, staged FURS procedure was performed 4 weeks later. The indwelling double-J stent was removed under local anesthesia 2 to 3 weeks from the last procedure. Stone-free status was determined by non-contrast computed tomography or KUB combined ultrasonography 4 weeks after the last FURS treatment. We defined stone clearance as no fragments or fragments less than 3 mm on standard radiograph. Patients who converted to PCNL or extracorporeal shock wave lithotripsy (ESWL), or those refused further therapy with significant residual fragments, were regarded as FURS treatment failures. All patients were followed up for at least 6 months to assess stone free status and renal function. For patients with complications, corresponding treatment effect was also concerned. When hydronephrosis was revealed by ultrasonography, intravenous pyelography, or retrograde pyelography was performed to evaluate potential ureteric stricture. Operating time was defined as the minutes from insertion of the ureteroscope to the completion of ureteral stent placement, and for staged procedures it was recorded as a total. The duration of hospital stays was the time from the day of operation until discharge, which was also recorded as a total for staged procedures. For patients who underwent more than 1 session, we also calculated the total time duration of therapy.

Statistical analysis was performed with SPSS 21.0 software. Data were expressed as mean ± standard deviation. We used the Student *t* test for continuous variables and the Chi-Squared test for categorical variables. *P* < .05 was considered as statistically significant.

## 3 Results

A total of 363 procedures were performed for 251 patients presented with 257 renal stones 2 cm or greater. Indications for FURS treatment included patient preference (28.7%), complex comorbidities such as bleeding diathesis, chronic anticoagulation and severe kyphoscoliosis (16.7%), failed prior PCNL (10.8%), solitary kidney (3.2%), and none indicated (40.6%). Patients and kidney stone characteristics were summarized in Table 1. Mean stone diameter was 26.5 mm (range 20–80 mm). These kidney stones were mainly multiple (75.5%) and located in mixed calices and renal pelvis (49.4%). Among the 127 stones located in mixed calices, 79.5% were involved in lower calyx location. Infrared spectroscopy was performed in 229 patients with available stone samples and the majority were composed of 2 or more stone types (45.9%). Calcium oxalate monohydrate (21.8%) and uric acid (9.6%) were most commonly identified in patients with stones of a single component.

**Table 1:**
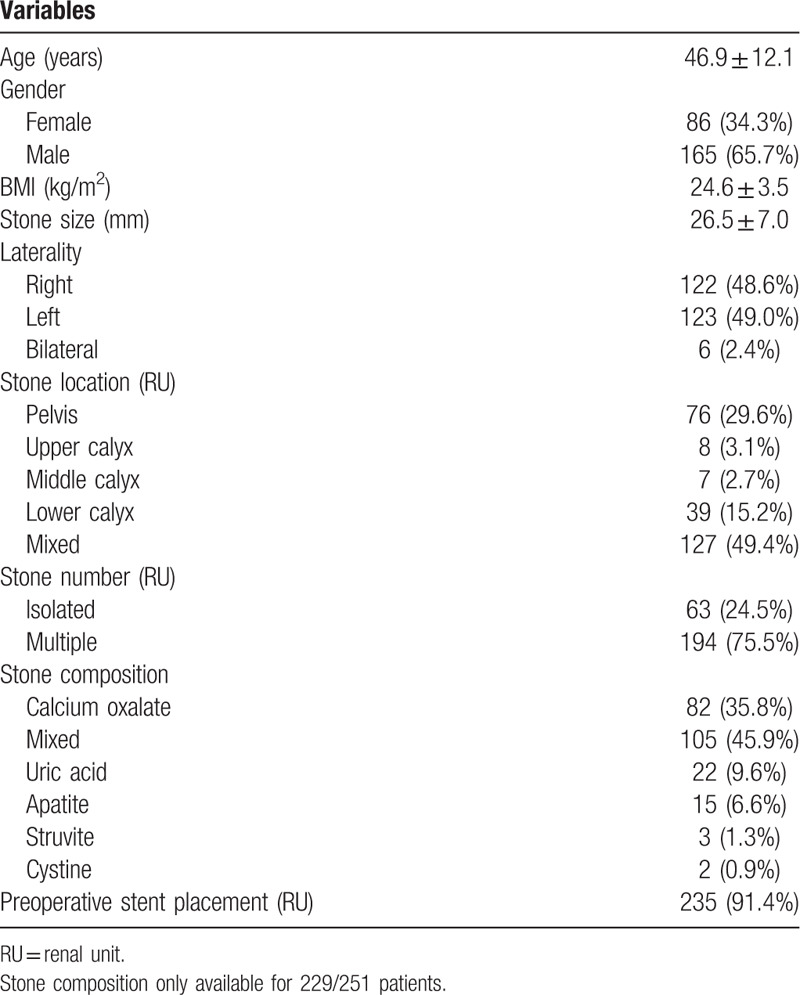
Patients and renal stone characteristics.

A total of 225 patients (230 renal units) were confirmed as stone free status after single or staged FURS treatment. Among the 26 patients (27 renal units) with significant residual fragments, 5 patients converted to PCNL, 13 converted to ESWL, and 8 refused further therapy, which were all regarded as FURS treatment failures. The mean number of procedures was 1.4 (range 1–5). The mean total operating time was 126.8 minutes (range 30–595 minutes). The SFR at the end of the first, second, and third procedure was 61.9%, 82.9% and 89.5%, respectively. Table 2 presented the stone clearance rates based on stone size. As stone size grows, the final SFR decreased and patients were more likely to receive more than 1 operative procedure. We also evaluated the outcomes based on stone location (Table 3), and the lowest clearance rates were observed in lower calyx calculi (87.2%) and multiple calyx calculi (83.5%).

**Table 2:**
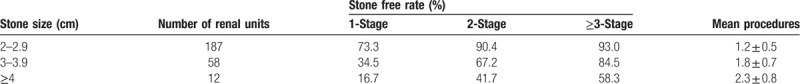
Stone clearance evaluation based on stone size.

**Table 3:**
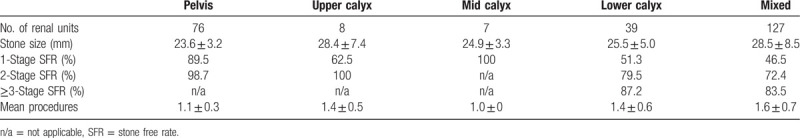
Comparison of stone clearance based on stone location.

The mean total postoperative stay was 3.5 days (range 1–24 days). The mean total time duration of therapy in patients who underwent more than 1 session was 41.0 days (range 30–127 days). Intra-operatively or early postoperative complications were listed in Table 4. The overall complication rate was 15.1%, and the most common complication was postoperative fever. Intraoperative minimal ureteral perforation was observed in one patient, which was managed with postoperative double-J stenting for 30 days. Five patients developed sepsis and 1 progressed to septic shock requiring intensive care. These patients were conservatively treated with appropriate antibiotics and recovered well. Two patients represented temporary hematuria postoperatively, which was spontaneously resolved. Steinstrasse formation occurred in 1 patient and was managed by rigid ureteroscope with no major sequences. One patient required blood transfusion, owing to postoperative coagulation disorders induced by urosepsis. There were 3 hospital re-admissions approximately 5 days after surgery due to postoperative fever. Over a period of 6-month follow-up, no ureteric stricture was identified.

**Table 4:**
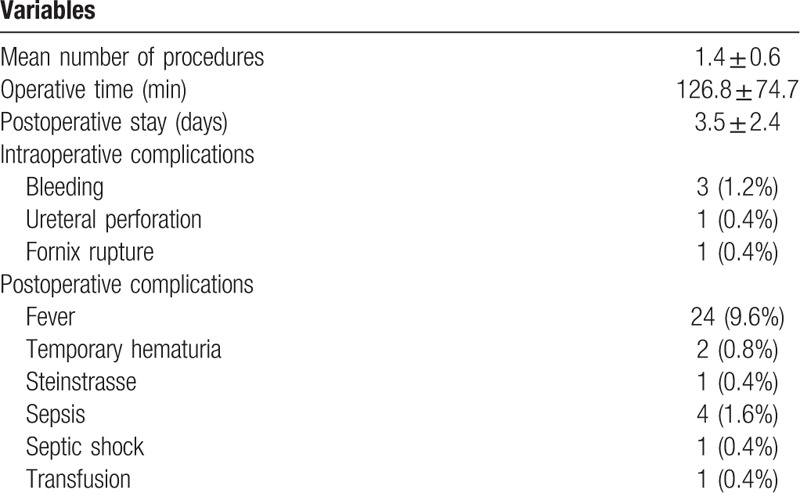
Intraoperative and postoperative results.

## 4 Discussion

The recent EAU guideline recommend PCNL as the first treatment option for renal stones larger than 2 cm in adults.^[1]^ However, the puncture and dilation procedures during PCNL have continued to be associated with a risk of bleeding, requirement for blood transfusion, or even arterial embolization. In addition, renal parenchymal damage is inevitable in the process of PCNL, leading to potential kidney function loss, especially in patients receiving multiple percutaneous procedures. To allow healing of the nephrostomy tract, daily routines and work of postoperative patients are also restricted for a certain period of time.

Performed through the natural orifice, FURS offers several potential advantages. Theoretically, it can avoid irreversible loss of renal parenchyma and significantly reduce the risk of severe bleeding.^[5]^ Thus, FURS is especially appropriate for nephrolithiasis patients emphasizing renal parenchyma preservation (such as solitary kidney), with bleeding diathesis or on chronic anticoagulation. Moreover, due to its excellent deflection, FURS allows access to all renal calices and shows advantages in the management of multiple kidney stones and renal sinus cysts.^[6,7]^ Furthermore, FURS also has short hospitalization and few restrictions on work or daily routines.

As early as 1998, Grasso and colleagues reported the outcomes of large renal stone patients receiving retrograde ureteropyeloscopic treatment with an overall SFR of 93.0% and complication rate of 5.9%.^[8]^ Subsequent publications supported this primary result and demonstrated similar successful management in ever larger cohorts (Table 5). According to a recent meta-analysis, the initial SFR was 71.2% in the FURS treatment of renal stones sized 2 to 3 cm, and the final SFR was 89.4% with an average of 1.4 procedures.^[12]^ As far as we know, there were only 2 prospective studies that compared the outcomes between FURS and PCNL in handling renal stones greater than 2 cm.^[13,14]^ Both studies showed that FURS with holmium laser lithotripsy could achieve a final SFR comparable to that of PCNL, indicating FURS was a feasible option for the treatment of large renal stones.

**Table 5:**
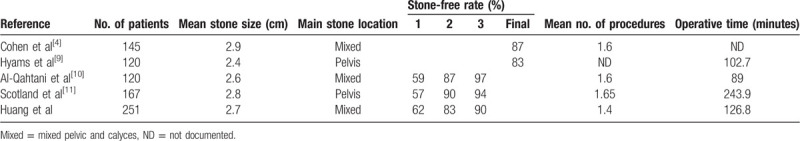
Stone characteristics and outcomes in large cohorts.

Up to our knowledge, this is the largest single-institution series of patients with renal stones greater than 2 cm treated by FURS with holmium laser. Our results showed that the initial SFR was 61.9%, which was inferior to previously published reports of PCNL for large renal stones. We think it may partly due to the deliberately limited operation time (within 90 minutes), with an attempt to lower the risk of infectious complications. In addition, the majority of our patients were with stones located in lower calyx (54.5%), which was intractable in retrograde endoscopic treatment. Despite this unfavorable factor, we achieved a final SFR of 89.5%, which is comparable to recent series by other groups and those achieved by PCNL.^[11,14]^ Furthermore, in this study, 26 patients chose PCNL/ESWL or refused any further therapy after single or staged FURS, which were all regarded as FURS treatment failure. The actual SFR should be a little higher if these patients accepted subsequent FURS procedures.

In the previous literature, stone clearance rates of FURS treatment were found to be mainly influenced by stone location and size. Renal stones located in the lower calyx may hamper the manipulation of ureteroscopy, leading to a significantly lower stone clearance rate or even treatment failure.^[15]^ Meanwhile, stone size was identified as an independent predictor of clinical outcomes of FURS treatment for large kidney stones.^[10,16]^ It is not surprising since the stone volume increases exponentially as the diameter of a calculi increases. In this study, the SFR of lower calyx stones was significantly lower than that of other calices or renal pelvis, and the SFR for stones larger than 4 cm was only 58.3% after an average of 2.3 procedures. Thus, we agree with the proposition that currently renal stones larger than 4 cm is not suitable for staged FURS, and PCNL should be considered preferentially.^[5]^

We have some experience to share in the FURS treatment of large renal calculi. First, we routinely used 13/15F ureteral access sheath in FURS procedures. The access sheath protects the ureter from repeated insertion of the scope, facilitates stone fragment extraction, enables continuous drainage flow to maintain good vision and low intrarenal pressure, and extends the longevity of ureteroscopes.^[17,18]^ Compared to the conventional access sheath (such as 12/14F), the larger diameter of 13/15F sheath allows more efficient fragment retrieval and better outflow. Second, most patients in this study were DJ-stented preoperatively to widen ureters by passive dilation, which made the placement of ureteral access sheath easier and safer. DJ-stenting was also reported to be associated with higher SFR and lower complication rates.^[19]^ Third, if we cannot finish FURS within 90 minutes, a staged operation was considered. In that case, stones easier to access were preferentially fragmented and extracted as thoroughly as possible, and the rest stones were just left for next procedure. Due to the reduced stone burdens, good vision was obtained in the following procedures and stone clearance is easier to achieve even in low calyx. Last but not least, we typically displace lower calyx stones into renal pelvis or other calices as long as technically feasible, which facilitated the stone fragmentation and extraction. Fragmentation or dusting was decided feasibly in the operation according to the hardness of stone. In most cases, stones were fragmented to appropriate size and extracted by the baskets.

Complications associated with FURS are not well documented and most typically consist of fever, temporary hematuria, and urinary tract infection. In early studies, the complication rate of FURS treatment for renal stones greater than 2 cm was 10.1%, with a major complication rate of 5.3%.^[20]^ A recent meta-analysis showed an overall complication rate of 16.1% in FURS treatment for renal stones sized 2 to 3 cm, with major complications occurred in 3.2% of patients.^[12]^ Moreover, the major complication rates associated with FURS had significantly decreased since May 2011 compared to the period between 1990 and 2011 (1.48% vs 5.01%), which may be related to the awareness of FURS complications, and the improvement of both surgery skill and endoscopy technology.^[21]^ Until now, blood transfusion has not been reported in previous studies treating large renal stones with FURS. However, we think the actual complication rate may be underestimated since severe complications tend to not to be reported.

In the present series, the overall complication rate was 15.1%, and the major complication rate was 3.6%. Several major complications such as ureteral perforation, steinstrasse, and urosepsis were recognized, which were all managed appropriately. It is worth noting that 1 patient required transfusion in this study due to the postoperative coagulation disorders induced by sepsis, which is seldom reported previously. Sepsis is the most dangerous complication during FURS treatment, contributing to 2 thirds of mortality associated with FURS.^[22]^ Large stone size, associated with great bacteria burden and long operative time, is found to be significantly associated with postoperative urosepsis.^[23]^ Increased intrarenal pressure during FURS procedures had also been demonstrated to be linked to sepsis.^[24]^ Thus, apart from appropriate prophylactic antibiotic use, we advocate the strategy that limit the operative time within 90 minutes and maintain a low intrarenal pressure during each FURS session, which was supported by several studies.^[25,26]^

The primary limitation of this study is the retrospective nature and selection bias. Moreover, the postoperative modalities for assessing SFR were not uniform and the majority of patients underwent KUB with ultrasonography. While non-contrast computed tomography is more appropriate to evaluate the stone status, cost, and logistical reasons greatly prohibit its routine use in China. A further limitation is the relative short follow-up time, therefore potential long-term complications such as ureter stricture were not assessable.

## 5 Conclusion

In conclusion, our results showed FURS could provide a comparable final SFR with acceptable complication rate in the treatment of large renal stones. We advocate FURS as a practical treatment option for the renal stones sized 2 to 4 cm, while PCNL may be a favorable option for patients with renal stones >4 cm currently. In the future, technological advances and experience increases may further extend the indication for FURS treatment in patients with large renal stones.

## Author contributions

**Conceptualization:** Jian-Sheng Huang, Jing Xie, Kefeng Xiao.

**Data curation:** Jian-Sheng Huang, Jing Xie.

**Formal analysis:** Jing Xie, Xiang-Jiang Huang, Qian Yuan, Hong-Tao Jiang.

**Methodology:** Jian-Sheng Huang, Xiang-Jiang Huang.

**Software:** Xiang-Jiang Huang, Qian Yuan.

**Supervision:** Hong-Tao Jiang, Kefeng Xiao.

**Writing – original draft:** Jing Xie.

**Writing – review & editing:** Jian-Sheng Huang, Qian Yuan, Hong-Tao Jiang, Kefeng Xiao.
